# Mpox in Young Woman with No Epidemiologic Risk Factors, Massachusetts, USA

**DOI:** 10.3201/eid2904.221921

**Published:** 2023-04

**Authors:** Mark J. Siedner, John Trinidad, Cesar G. Berto, Catherine M. Brown, Lawrence C. Madoff, Ellen H. Lee, Maryam Iqbal, Olivia Samson, John Albin, Sarah E. Turbett, Olivia Davies, Daniela Kroshinsky, David Hooper, Elizabeth Hohmann, Kevin Ard, Erica S. Shenoy

**Affiliations:** Masschussetts General Hospital and Harvard Medical School, Boston, Massachusetts, USA (M.J. Siedner, J. Trinidad, C.G. Berto, J. Albin, S.E. Turbett, O. Davies, D. Kroshinsky, D. Hooper, E. Hohmann, K. Ard, E.S. Shenoy);; Massachusetts Department of Public Health, Boston (C.M. Brown, L.C. Madoff);; New York City Department of Health and Mental Hygiene, Long Island City, New York, USA (E.H. Lee, M. Iqbal, O. Samson)

**Keywords:** mpox, monkeypox virus, viruses, case investigations, clinical epidemiology, California, New York, Massachusetts, United States

## Abstract

We describe a case of mpox characterized by a circularly distributed facial rash but no identified risk factors. Fomite transmission of monkeypox virus from contaminated linen at a massage spa was suspected. Clinicians should consider mpox in patients with consistent clinical syndromes, even in the absence of epidemiologic risk factors.

During the 2022 global outbreak, ≈95% of mpox cases, caused by monkeypox virus infection, were attributed to close physical contact, and >98% were reported among men (*1*,*2*). We describe a case of a young woman who had no sexual or close physical contact with anyone suspected of having mpox during the 2 months before she had a confirmed monkeypox virus infection.

A woman in the United States in her late 20s, who had hypothyroidism after curative thyroidectomy for medullary thyroid cancer 7 years before, sought care in July 2022 at a hospital emergency department 8 days after a facial rash developed. The rash was initially pruritic, and erythematous macules were located on the bilateral infraorbital and malar areas, lower cutaneous lip, and chin, which progressed to vesicles followed by pustules. She was prescribed doxycycline and valacyclovir. She experienced subjective fevers, myalgias, bilateral cervical lymphadenopathy, and scattered papules that developed bilaterally on her legs and arms, prompting her to return to the emergency department (Figure). She also had tender cervical lymphadenopathy and scattered erythematous macules on her limbs. Laboratory tests were negative for HIV, syphilis, gonorrhea, *Chlamydia* sp., herpes simplex virus, and varicella zoster virus. PCR for orthopoxvirus was positive and had a cycle threshold of 21.2. The patient was started on tecovirimat. Facial swelling and lymphadenopathy resolved within the next 48 hours, and no new lesions were noted thereafter (Figure).

**Figure:**
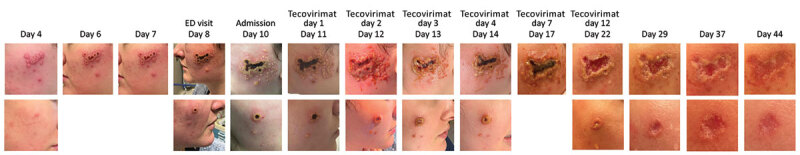
Progression of facial rash during mpox in a young woman in the absence of epidemiologic risk factors, Massachusetts, USA. Days since rash onset or beginning tecovirimat therapy are indicated. The rash began with pruritic erythematous macules on the bilateral infraorbital and malar areas, lower cutaneous lip, and chin and, by day 4, had progressed to vesicles followed by pustules on day 6 (top row, left cheek; bottom row, right cheek). On day 8 after rash onset, the patient had multiple confluent ulcers; macerated rolled borders were observed on the left cheek, and a single, large, deep-seated ulcer that had raised borders and a central hemorrhagic crust was observed on the right cheek. Satellite blisters and papules were present at early stages of ulcer development. The patient was started on tecovirimat on day 11 after rash onset, after which her lesions continued to evolve and had eventual loss of central eschar but persistent exudative, macerated borders by day 12 of tecovirimat therapy (day 22 after rash onset). Smaller lesions were treated with mupirocin ointment and dressed with loose gauze coverings. Toward the end of her 14-day treatment course (day 22), the escharotic ulcers developed granulated tissue. Ulcers had abundant granulated tissue and no central eschar and had begun to reepithelialize ≈2 weeks after completion of therapy (day 37).

The patient resided alone in New York and had traveled to California and Massachusetts for business and leisure during the 3 weeks before her rash developed. She described herself as a woman who has sex with men only. She reported no sexual activity or any close intimate contact with anyone during the 3 months before her rash developed and had no contact with anyone suspected of having mpox disease. She had a history of acne but had not used new skin products in the preceding weeks. She reported receiving 2 massages in the preceding weeks, 1 at a hotel spa 13 days before rash developed and another at a private day spa 4 days before rash developed. On both occasions, she laid face down on a massage table on top of a circular pillow covered by thin linen or a towel. She had a dentist appointment 6 days before and a dermatologist appointment 3 days before rash developed. On both occasions, the clinicians donned clean disposable gloves before contact.

Because of the physical location of the patient’s lesions and lack of sexual encounters during the incubation period, an ensuing public health investigation focused on the spa visits. No other mpox cases among staff or clientele of either spa were identified during a review of cases by the New York City Department of Health and Mental Hygiene or Massachusets Department of Health or by matching staff and client lists with electronically reported mpox results (New York City Department of Health and Mental Hygiene only). Both spas reported that they changed coverings on the massage tables between clients, used freshly laundered linens and towels, and used a disinfectant that has efficacy against enveloped viruses. Environmental sampling at the spas was not performed because of the amount of time that had passed between the spa visits and mpox diagnosis. No mpox cases after visits to the dentist were identified.

We report an mpox case in a woman who had no epidemiologic risk factors for this disease. Although the transmission source in this case could not be confirmed, the rash locations and pattern suggest inoculation through fomites from contaminated facial towels or other linens, as has been reported for monkeypox virus and other poxviruses (*6*,*7*). In a cluster of 20 cases linked to a tattoo establishment, where monkeypox virus was recovered on piercing equipment (*5*), persons visiting the establishment were infected >2 weeks after the suspected index case, suggesting prolonged virus viability on surfaces. Surface contamination by viable monkeypox virus has also been reported in hospital rooms and community settings (*8*). Viable virus is more recoverable from porous materials, such as linens and towels, than nonporous materials, such as metals and plastics (*9*). The Centers for Disease Control and Prevention provides comprehensive sterilization recommendations for both linens and hard nonporous materials (*10*). The rash in this case was characterized by large deep wounds that did not begin to granulate until ≈5 weeks after rash onset, indicating the need to elucidate viral shedding duration from these types of ulcers. 

In conclusion, as in recent reports of persons who had mpox without intimate contact (*3*–*5*), this case highlights the importance of maintaining clinical suspicion of mpox for persons who do not meet known epidemiologic criteria. This case also supports the possibility of fomite-based transmission of monkeypox virus. 
